# Evaluation of five fixation methods for mandibular sagittal split osteotomy in a significant advancement with counterclockwise rotation: a finite element study

**DOI:** 10.4317/medoral.27600

**Published:** 2025-10-14

**Authors:** Gabriel Cury Batista Mendes, Gustavo Batista Grolli Klein, Constantinos Laskarides, Archana Viswanath, Beethoven Estevao Costa, Osvaldo Magro-Filho, Maísa Pereira-Silva, Paulo Domingos Ribeiro-Junior

**Affiliations:** 1 Department of Oral and Maxillofacial Surgery, University of Sagrado Coração (USC), Bauru, São Paulo, Brazil; 2 Department of Dental Anesthesiology, University of Itaúna, Minas Gerais, Brazil; 3 Department of Oral and Maxillofacial Surgery, São Leopoldo Mandic - Campinas, São Paulo, Brazil; 4 Department of Oral and Maxillofacial Surgery, Tufts University, Boston, Massachusetts, USA; 5 Department of Diagnosis and Surgery, School of Dentistry, São Paulo State University (UNESP), Araçatuba, São Paulo, Brazil

## Abstract

**Background:**

This study aimed to evaluate the biomechanical behavior of five different fixation methods used in bilateral sagittal split osteotomy (BSSO), focusing on their performance with miniplates and monocortical screws during 10-mm advancement and 20º counterclockwise rotation of the occlusal plane.

**Material and Methods:**

A three-dimensional model of a human mandible, derived from computerized tomography scans and including all teeth except the third molars, was utilized. The BSSO procedure was simulated using SolidWorks 2017 CAD software (Dassault Systemes, SolidWorks Corp, USA) according to the techniques outlined by Epker. Five fixation models were tested: Model M1, one straight 4-hole miniplate with four monocortical screws; Model M2, two straight 4-hole miniplates with eight monocortical screws; Model M3, one 10-hole double miniplate with two bridges and ten monocortical screws; Model M4, one 8-hole 20º angled double miniplate with two bridges and eight monocortical screws; and Model M5, one semi-curved 6-hole miniplate with six monocortical screws. Each model was subjected to two loading patterns: 100 N posteriorly and 50 N anteriorly. The biomechanical performance was analyzed qualitatively and quantitatively, focusing on the bone, screws, and plates.

**Results:**

Models M1 and M3 exhibited the poorest biomechanical stability; Model M2 demonstrated the highest stability; and Model M5 showed the best load distribution.

**Conclusions:**

A model using two straight 4-hole plates offers more stable osteosynthesis, whereas a semi-curved plate with six nonlinear screws ensures effective load distribution with reduced stress concentration.

## Introduction

Biomechanical studies have shown that bone segment rigidity with bicortical screw fixation tends to be greater than that achieved with miniplates and monocortical screws (1 - 3). However, some disadvantages of bicortical screw fixation have also been reported, such as an increased possibility of condylar torque, damage to nerve structures, and injury to teeth adjacent to the fixation site (1 , 4 , 5).

Despite the greater mechanical resistance associated with bicortical screw fixation, the use of monocortical plates and screws offers several advantages, such as removing the need for transbuccal access, allowing correct manipulation of the proximal segment for correct condylar positioning, causing fewer difficulties in positioning bicortical screws in cases of large advancements due to reduced bone contact among the segments, and showing less probability of damage to the inferior alveolar nerve (4). Moreover, a recent systematic review and meta-analysis found no statistically significant difference in skeletal stability between the use of bicortical screws and miniplates with monocortical screws (6), although some studies have reported increased long-term relapse in patients treated with bicortical screws instead of miniplates (4).

The stability of orthognathic surgical procedures is also influenced by the type of movement. Mandibular advancement is commonly associated with higher relapse rates (7 , 8), and patients with mandibular advancement &gt;10mm tend to show higher rates of postoperative instability (9 , 10). The amount of advancement, type of fixation material, and manipulations of the occlusal plane are considered the main factors that influence surgical relapse (4) and anti-clockwise rotation of the occlusal plane is considered the most unstable movement (11 , 12). If the fixation material is not strong enough, postoperative relapse may occur (13). Maxillomandibular advancement associated with counterclockwise rotation of the occlusal plane has been widely recommended for patients with obstructive sleep apnea (14 , 15).

This study aimed to evaluate different methods of fixation for sagittal split ramus osteotomy (SSRO) using miniplates and monocortical screws with large advancements (10mm) and 20° counterclockwise rotation of the occlusal plane under masticatory loads applied to the posterior and anterior regions of the mandible. This evaluation was performed using the finite element method to assess bone segment displacement and load distribution throughout the osteosynthesis system.

## Material and Methods

Mandibular finite element modeling

**Figure 1:**
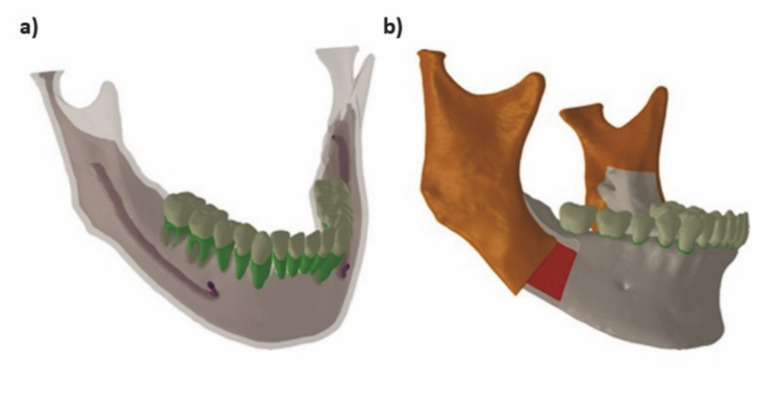
a) Image of the semi-transparent mandible model from the computed tomography. b) 3D SSRO with a 10-mm advancement and 20º rotation.

A three-dimensional model of a human mandible was manufactured from computerized tomography images showing all teeth, except the third molars, extracted from an image bank (Figure 1).

1

To standardize the mandibular support zone, structures were created in the insertion regions of the temporal, masseter, and medial pterygoid muscles. Two structures were created to standardize the load application area: one anterior in the region of teeth 31, 32, 41 and 42, and one posterior in the region of teeth 45, 46 and 47. The posterior loading intensity was 100 N, and the anterior loading was 50 N, both with the vector perpendicular to the occlusal plane.

Osteotomy design

**Figure 2:**
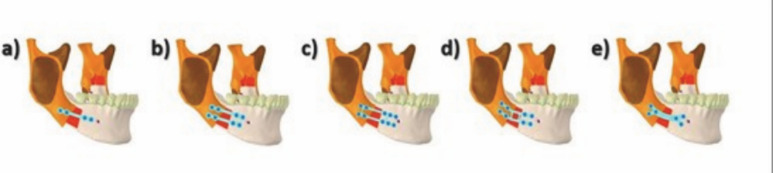
Final geometric figures of all five different models.

Using the SolidWorks 2017 CAD software (Dassault Systemes, SolidWorks Corp, USA), the SSRO procedure was performed bilaterally according to the technique described by Epker (16), and was followed by a 10-mm advancement and 20° counterclockwise rotation of the occlusal plane (Figure 2). Through analysis and detailed measurements using digital calipers (Mod. 500-196-30B; Mitutoyo Sul Americana Ltda., Suzano, Brazil) and a digital microscope (B008; Supereyes, Shenxhen D &amp; F, Ltd, Bantian Village, China) with magnification of 10x-500x, the fixation models (plates and screws) were rebuilt using SolidWorks software (Figure 1).

2

Internal fixation techniques

Five different types of osteosynthesis were used with monocortical plates and screws from different commercially available brands with specific manufacturer indications for use in SSRO fixation. These models were adapted bilaterally in the regions of the osteotomies, in accordance with the descriptions provided below (Figure 2):

Model M1: A straight plate with four holes and four monocortical screws (KLS Martin, Tuttlingen, Germany)

Model M2: Two straight plates with four holes each and eight monocortical screws (KLS Martin, Tuttlingen, Germany)

Model M3: One 10-hole dual plate with two bridges and 10 monocortical screws (Jeil Medical Corporation, Seoul, South Korea)

Model M4: One double-bore 8-hole dual plate angled at 20º with two bridges and eight monocortical screws (Traumec Health Technology, Rio Claro, Brazil).

Model M5: A semi-curved plate with six nonlinear holes and six monocortical screws (NeoOrtho, Curitiba, Brazil). All screws used were 5mm in length.

Finite element analysis

All models were exported from the SolidWorks software to the Ansys Workbench V18.2 finite element simulation software (Ansys Inc., Canonsburg, PA, USA) through the Ansys import supplement. The moduli of elasticity and Poisson's coefficients of the materials were derived from the literature (Table 1). The plates were simulated as titanium grade 2, and the screws were simulated as titanium grade 5 (Ti-6Al-4V). All the structures were considered isotropic, homogeneous, and linearly elastic.

Table 1

**Figure 3:**
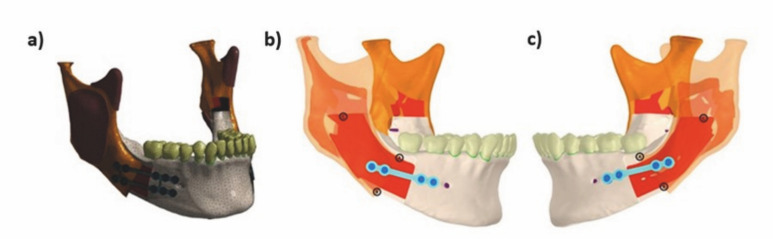
a) Image of the meshes used. b) Points A, B, and C selected for measurement of displacement between bone segments on the right side - working side. c) Points A, B and C selected for measurement of displacement between bone segments on the left side - balance side.

A finite element mesh (Figure 3) was generated with tetrahedral quadratic elements of 10 knots (solid 187), and the numbers of knots/elements ranged from 1577586/950470 to 1997778/1202081. All the models were then solved (Windows 10 64-bit, Intel I7 6800k processor, and 112 GB RAM). Graphical and numerical plots of the data were recorded, evaluated and compared.

3

The results were recorded, evaluated, and compared graphically and numerically for qualitative and quantitative analyses. Three aspects were analyzed: bone, screws and plates.

For evaluation of bone, the displacement and distance of the proximal and distal stumps were considered because the intensity of the micro-movement between the segments could affect the bone-repair process. To verify the stability of the fixation method, three points in the proximal segment (A, B, and C) (Figure 3) where the distal segment showed initial contact were selected to measure the displacements among the bone surfaces. After the load application generated a le displacement, the distance between these points and the region of initial contact in the distal segment was measured. The values of peak displacement (mm) in the posterior and anterior loads, both on the working (right) and balancing (left) sides of the mandible, were evaluated. The points (A, B, or C) at which the displacement peak occurred were recorded and model M1 was used as the control to facilitate comparison.

The screws and plates were analyzed using the von Mises criterion because of the ductile characteristics of titanium. The results were also considered to be proportional to the yield limits of 880 MPa for grade five titanium (screws) and 410 MPa for grade two titanium (plates).

## Results

The values of the displacement peaks (in mm) between the bone surfaces under the posterior and anterior loads were evaluated in the five models, and are listed in Table 2.

Table 2

**Figure 4:**
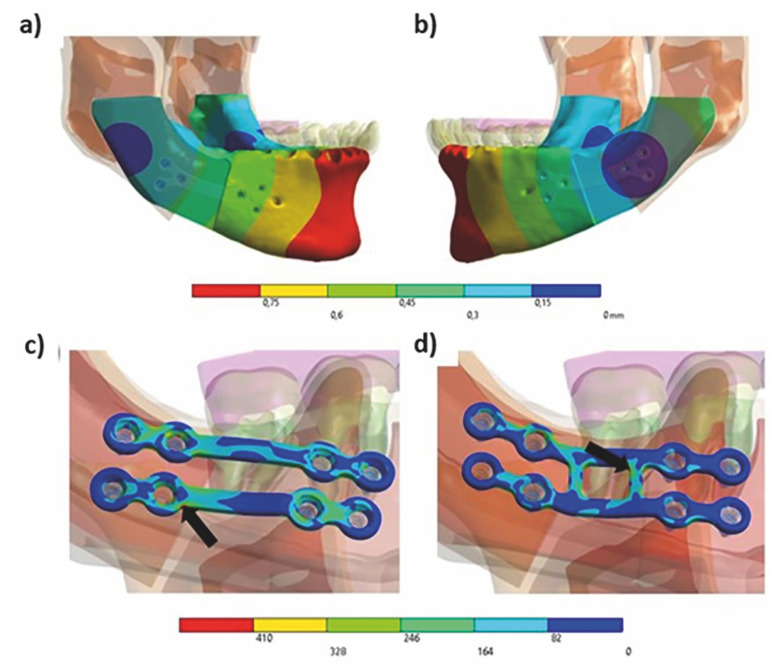
a) Displacement of the bone segments after posterior loading on the right side-working side. b) Displacement of the bone segments after posterior loading on the left side-balance side. c) Locations of High Stress Concentrations (indicated by black arrows). M2 Model: High stress peak identified within the link region; d) M4 Model: High stress peak observed in the connection bridge area.

When the rotational movement of the mandible was evaluated, all models showed lateral movement of the mandible body, although the radius of rotation varied significantly (Figure 4) and the space formed between the bone surfaces also accounted for the displacement results. Under the anterior load, all models showed rotational movement without lateral displacement, with both sides turning similarly and the center of rotation in the posterior region of the plates.

4

The maximum stress values on the screws using the von Mises criterion are listed in Table 3. Qualitatively, the stresses were distributed on the screws according to the rotational movement, with concentrations in the upper or lower regions of the screws according to their location relative to the flexion of the plates. The maximum voltage magnitudes occurred in the posterior screws, indicating greater flexion of the plates in that region. The maximum tension values at the posterior and anterior loads on the plates are shown in Table 4.

Table 3 Table 4

Voltage peaks occurred at the angles formed between the metal rings surrounding the screws and the metal bars that connected these rings, especially in the region of the screws closer to the osteotomy of the proximal stump. An exception to this behavior was observed in model M4, where the peak developed at one of the angles formed in the union bridges of the plate (Figure 4).

## Discussion

Numerous studies have investigated the biomechanics of SSRO osteosynthesis methods. However, knowledge of these dynamics in movements involving large advancements with counterclockwise rotation of the occlusal plane is limited, particularly because of the small number of studies addressing this topic. Most studies on this topic have preferred in vitro testing (1 , 2 , 10 , 17 , 18). More recently, finite element analysis (FEA) has become an interesting and widely used tool for biomechanical studies of the mandible and has been validated in several studies (3 , 19 , 20). FEA allows the analysis of various parameters such as stress, tension, and displacement, which are difficult to evaluate using other methodologies (21). However, studies using FEA to evaluate SSRO fixation methods in cases involving large advancements and counterclockwise rotations are still lacking in the current literature.

To evaluate the bone displacement in the five tested groups, the peaks of the bone segment movement were recorded. The amount of displacement that can impair the repair process depends on the individual biological and mechanical characteristics of each patient. However, this type of analysis allows prediction of the models that show a higher risk of interfering with the expected bone repair. Importantly, movements that do not necessarily generate distancing of the bone segments, such as sliding, can be equally deleterious, reinforcing the relevance of analyzing all segment motions and not just evaluating gap formation.

Vajgel et al. (22) suggested evaluating the amount of gap generated between the bone segments under loading to determine which osteosynthesis method provides greater rigidity and, therefore, a lower probability of adversely affecting bone repair. Certain threshold values for gap formation remain controversial. A gap of 150 m (0.150mm) has been reported as the biological limit for conditions that predispose patients to non-union or pseudoarthrosis (22). However, Jagodzinski et al. (23) demonstrated that while micromovements of 0, 2 and 1mm could facilitate the formation of bone callus during fracture healing, excessive movements could lead to relapse.

Load values of 100 N in the posterior region and 50 N in the anterior region were adopted to simulate masticatory forces during the early postoperative period, based on a previous study reporting an average masticatory force of 66 N in the first two weeks and 128 N in the fourth postoperative week in patients undergoing SSRO (24).

Models M1 and M3 exhibited lower results in the displacement evaluation in the posterior load test, with a difference of only 2%. In a comparison of models M1 and M2, the use of only one 4-hole plate per side in model M1 clearly resulted in lower performance. In model M3, the reduced plate thickness (0.8mm instead of 1.0mm) likely contributed to its inferior performance. Model M5, which also had a single plate, exhibited better biomechanical behavior than model M1. The posterior load displacement was 56% lower and the anterior load was 30% lower in model M5, possibly owing to differences in design and robustness. Similar results supporting the use of plates similar to model M5 for large advancements with counterclockwise rotation have been reported in the literature (1 , 25 , 26), although Peterson et al. (27) reported contrasting findings, likely due to design differences.

The displacement peaks were higher under the anterior load than under the posterior load, likely due to the lever effect created by the distance between the load application point and the center of resistance. This highlights the harmful potential of anterior loading on segment stability (27), especially since the anterior load was only half the intensity of the posterior load.

The models showing the highest and lowest peak displacements were M1 (1.4094mm) and M2 (0.4813mm), respectively. The second-highest displacement was observed in model M5 (0.9926mm), where the presence of only one screw near the osteotomy likely provided less support against rotation (plate flexion) in that area.

When analyzing the overall segment displacement under both posterior and anterior loads on both the working (loaded) and balancing (nonloaded) sides, model M1 showed the highest displacement, confirming that it was the least stable system. In contrast, model M2 (with an additional identical plate) demonstrated a significant improvement: 41% displacement on the work-side shift under a posterior load, 31% less displacement at the working side under a posterior load, and 66% less shift bilaterally under an anterior load. Thus, the system with two 4-hole straight plates (model M2) was the most stable. Similar results have been described previously (1 , 11 , 26 , 28 , 29), supporting the stability of SSRO fixation using two straight 4-hole plates.

An alternative to the use of two straight plates is a bridged double-plate design that connects the upper and lower segments. A recent study (30) evaluated the effects of such bridges using biomechanical testing, photoelastic analysis, and FEA to compare three configurations: two separate 4-hole plates, an 8-hole double plate with one connecting bridge, and an 8-hole double plate with two connecting bridges. The best mechanical resistance and stress distribution were observed with the two separate plates, whereas the bridged plates exhibited less displacement. In that study, only posterior loading was simulated, and the results were similar to those found in the present study, wherein model M4 showed smaller displacements than model M2 under posterior load. However, for the loads in the anterior region, the displacement in model M2 was considerably lower than that in model M4.

A noteworthy difference in bridge design is the location of the bridges. In model M3, the bridges were positioned in the region of the screws, whereas in model M4, the bridges were located in the intermediate region of the plate, closer to the region of the application of subsequent loads. This difference likely contributed to the better performance of model M4, since the bridges in the high-stress regions may have acted as support structures.

The compressive force in the bone around the fixation system screws has already been used as a parameter to evaluate the tendency of the system to trigger bone resorption around the screw, leading to loosening and possible osteosynthesis failure (22). However, the process of bone resorption is generally slow and more likely to occur in the later healing phases when mechanical demands are reduced. Therefore, in the present study, we focused on the possibility of structural changes to the titanium screws and plates subjected to loadings to determine the possibility of important structural changes threatening the success of osteosynthesis.

In model M1, under a posterior load, the tension peaks on the screws exceeded the yield strength limit of grade 5 titanium. Thus, under these conditions, this load-loaded system showed a greater potential to generate plastic deformation in screws, indicating a risk to the success of osteosynthesis. However, the performance of the plates, even in this group, was much lower, indicating less relevance of the screws to the risk of treatment failure. In other words, the plates tend to fail before the screws; therefore, assessment of the clinical impact of plate failure should be prioritized. Stringhini et al. (28) used the finite element method and observed a relationship between higher stress in the screws and less bone displacement in the segments. However, this relationship was not observed in the present study. A possible explanation for this is the use of locking plates, unlike the conventional plates used in the present study.

The stress concentration was higher in the screws near the osteotomy site, especially in the proximal segment, indicating greater plate flexion in this region. Torezan et al. (30) also reported that the mandibular ramus was a stress concentration area, even in mandibles without osteotomy. This factor should be considered when selecting the osteosynthesis hardware and screw placement.

With the exception of the plates in model M5, all plates exhibited tension peaks above the flow limit of titanium. Although a slight plastic deformation does not necessarily compromise treatment, these results represent plates subjected to risk treatment. Models M1 and M3 showed high values of maximum tension peaks that were two to three times higher than the limit of flow of titanium, implying the possibility of plastic deformation in the material if it was subjected to the simulated conditions. This behavior aligns with the observed inferior performance of thinner plates, especially the 0.8-mm plate used in model M3. These findings reinforce the recommendation for the use of thicker and more robust plate designs in clinical practice. Similarly, Ellis and Esmail (12) suggested that plates thicker than 1mm provide substantially improved mechanical strength and reduce the likelihood of plastic deformation under functional loads. Although the results provide a strong biomechanical basis for clinical decision-making, clinical studies are still needed to confirm the mechanical behavior and bone healing responses associated with these different fixation methods.

## Conclusions

The results of this study show that for SSRO fixation involving advancement and counterclockwise rotation, the use of two 4-hole straight plates provides greater osteosynthesis stability. The use of a semi-curved plate with six nonlinear screws also demonstrated good load distribution and reduced stress concentration. Conversely, fixation using a single 4-hole straight plate yielded poor mechanical performance.

## Figures and Tables

**Table 1:** Table Mechanical properties of materials used in simulation.

Material	Young's Modulus (GPa)	Poisson's Coefficient
Dentin (HOLMES et al., 1996)	18,6	0.31
Periodontal Ligament (REES & JACOBSEN, 1997)	0,05	0,45
Cancellous Bone (HOLMES et al., 1996)	1,37	0.3
Cortical Bone (HOLMES et al., 1996)	13,7	0.3
Titanium Grade 2 (MATWEB, 2016)	105	0,37
Titainum Grade 5 (MATWEB, 2016)	113,8	0,342

1

**Table 2:** Table Values of the displacement peaks (in mm) and the percentage of the result in relation to the peak of the control model (M1 peak = 100%).

	Posterior Load Right side	Posterior Load Left side	Anterior Load Right side	Anterior Load Left side
Model M1 (Martin)	0,7497 - 0,026 =0,7471 / 100% (A)	0,6794 - 0,001 =0,6793 / 91% (C)	1,4094 - 0 =1,4094 / 100% (C)	1,2083 - 0,002 =1,2081 / 85% (C)
Model M2 (2x Martin)	0,4489 - 0,029 =0,446 / 59% (A)	0,2094 - 0,001 =0,2093 / 28% (C)	0,4813 - 0 =0,4813 / 34% (C)	0,4367 - 0,0015 =0,4352 / 31% (C)
Model M3 (Jeil)	0,7619 - 0,05 =0,7614 / 102% (A)	0,3408 - 0,038 =0,337 / 45% (A)	0,6126 - 0 =0,6126 / 43% (C)	0,556 - 0,0015 =0,5545 / 39% (C)
Model M4 (Traumec)	0,2585 - 0,046 =0,2539 / 34% (B)	0,3622 - 0,001 =0,3621 / 48% (C)	0,8242 - 0 =0,8242 / 58% (C)	0,7062 - 0,0016 =0,7046 / 50% (C)
Model M5 (NeoOrtho)	0,3381 - 0,042 =0,3339 / 44% (A)	0,3976 - 0,001 =0,3975 / 53% (C)	0,9926 - 0 =0,9926 / 70% (C)	0,857 - 0,0017 =0,84 / 59% (C)

2

**Table 3:** Table Peak values of the results on the screws according to the von Mises criterion (in MPa) and its percentage in relation to the yield limit of titanium.

	Posterior Load Right side	Posterior Load Left side	Anterior Load Right side	Anterior Load Left side
Model M1 (Martin)	1209,2 / 137%	593,9 / 67%	771 / 87%	829,1 / 94%
Model M2 (2x Martin)	717,8 / 81%	272,3 / 31%	506 / 57%	440,5 / 50%
Model M3 (Jeil)	687 / 78%	580,2 / 66%	585,4 / 66%	771,7 / 87%
Model M4 (Traumec)	705 / 80%	283 / 32%	600,5 / 68%	486,4 / 55%
Model M5 (NeoOrtho)	713,7 / 81%	383,2 / 43%	533,7 / 60%	637,4 / 72%

3

**Table 4:** Table Peak values of the results on the plates according to the von Mises criterion (in MPa) and its percentage in relation to the yield limit of the titanium.

	Posterior LoadRight side	Posterior LoadLeft side	Anterior LoadRight side	Anterior LoadLeft side
Model M1 (Martin)	925 / 225%	661,8 / 161%	726,7 / 177%	1067,9 / 260%
Model M2 (2x Martin)	579,8 / 141%	375,6 / 91%	404 / 98%	540,6 / 132%
Model M3 (Jeil)	1479,2 / 361 %	825,6 / 201%	970,4 / 236%	1005,3 / 245%
Model M4 (Traumec)	511,1 / 124%	354,3 / 86%	536,9 / 131%	611,9 / 149%
Model M5 (NeoOrtho)	387,3 / 94%	232,7 / 57%	406,3 / 99%	375,9 / 91%

4

## Data Availability

Declared none.
